# Two-Year Monitoring of Water Samples from Dam of Iskar and the Black Sea, Bulgaria, by Molecular Analysis: Focus on *Mycobacterium* spp.

**DOI:** 10.3390/ijerph120707430

**Published:** 2015-06-30

**Authors:** Stefan Panaiotov, Ivan Simeonovski, Victoria Levterova, Ventzislav Karamfilov, Nadia Brankova, Kristin Tankova, Katrina Campbell, Pauline Jacob, Karim Helmi, Bas Boots, Emilio D’Ugo, Stefania Marcheggiani, Laura Mancini, Ulrich Breitenbach, Erik Mielke, Todor Kantardjiev

**Affiliations:** 1 National Center of Infectious and Parasitic Diseases, 1504 Sofia, Bulgaria; E-Mails: ivanos@dir.bg (I.S.); vikis@abv.bg (V.L.); nbrankova@abv.bg (N.B.); ktankova@abv.bg (K.T.); kantardj@ncipd.org (T.K.); 2 Institute of Biodiversity and Ecosystem Research, BAS, 1113 Sofia, Bulgaria; E-Mail: karamfilov.v@gmail.com; 3 Institute for Global Food Security, Queen’s University, Belfast BT9 5 AG, UK; E-Mail: katrina.campbell@qub.ac.uk; 4 Veolia Environnement Recherche and Innovation, Department Environnement Sante - Solutions d'Analyse Environnementale, 94410 Saint Maurice, France; E-Mails: pauline.jacob@veolia.com (P.J.); karim.helmi@veolia.com (K.H.); 5 UCD School of Biosystems Engineering, University College Dublin, Dublin 4, Ireland; E-Mail: b.boots@ucd.ie; 6 Istituto Superiore di Santia, 00161 Rome, Italy; E-Mails: emilio.dugo@iss.it (E.D.); stefania.marcheggiani@iss.it (S.M.); laura.mancini@iss.it (L.M.); 7 MARILIM Gesellschaft für Gewässeruntersuchung mbH Heinrich-Wöhlk-Str. 14 24232 Schönkirchen, Germany; E-Mails: breitenbach@marilim.de (U.B.); mielke@marilim.de (E.M.)

**Keywords:** molecular detection, PCR, monitoring pathogens, Bulgaria

## Abstract

The coast of the Bulgarian Black Sea is a popular summer holiday destination. The Dam of Iskar is the largest artificial dam in Bulgaria, with a capacity of 675 million m^3^. It is the main source of tap water for the capital Sofia and for irrigating the surrounding valley. There is a close relationship between the quality of aquatic ecosystems and human health as many infections are waterborne. Rapid molecular methods for the analysis of highly pathogenic bacteria have been developed for monitoring quality. Mycobacterial species can be isolated from waste, surface, recreational, ground and tap waters and human pathogenicity of nontuberculose mycobacteria (NTM) is well recognized. The objective of our study was to perform molecular analysis for key-pathogens, with a focus on mycobacteria, in water samples collected from the Black Sea and the Dam of Iskar. In a two year period, 38 water samples were collected—24 from the Dam of Iskar and 14 from the Black Sea coastal zone. Fifty liter water samples were concentrated by ultrafiltration. Molecular analysis for 15 pathogens, including all species of genus *Mycobacterium* was performed. Our results showed presence of *Vibrio* spp. in the Black Sea. Rotavirus A was also identified in four samples from the Dam of Iskar. Toxigenic *Escherichia coli* was present in both locations, based on markers for *stx1* and *stx2* genes. No detectable amounts of *Cryptosporidium* were detected in either location using immunomagnetic separation and fluorescence microscopy. Furthermore, mass spectrometry analyses did not detect key cyanobacterial toxins. On the basis of the results obtained we can conclude that for the period 2012–2014 no *Mycobacterium* species were present in the water samples. During the study period no cases of waterborne infections were reported.

## 1. Introduction

During the last two decades waterborne infections have contributed to the emergence of illnesses in Bulgaria, such as Q-fever and tularemia, which were previously rare or unknown [1,2,3]. Studies have confirmed that the sources of infections originated from the drinking water supply system. Contamination of water sources often occurs by accidental falls of animals into water reservoirs or contamination by fecal matter linked to agricultural activities. Possible microbial pollution along the Black Sea marine coastal zone, especially in sea resorts, is a serious ecological and public health concern. The Bulgarian Black Sea shore is a popular recreational and vacation destination for tourists. The coast is 378 km long of which 130 km are tourist beaches. Two large industrial seaports, Varna and Burgas exert anthropogenic pressure on the marine environment. The Dam of Iskar, with a capacity of 675 million m^3^, is part of the largest artificial lake in Bulgaria, and a popular place for aquatic leisure activities. Furthermore, the Dam of Iskar is the main source of tap water for the capital Sofia, electricity production and for irrigation of the surrounding valley.

There is a close relationship between the quality of aquatic ecosystems and human health. This relationship stems primarily from the consumption of water potentially being polluted by chemicals and/or contaminated by pathogenic organisms. Monitoring water quality is of paramount importance for prevention and safeguarding public health.

Although routine examination of drinking water and sea water for a number of chemical and microbiological parameters has been carried out by environmental protection authorities in Bulgaria, no published reports have evaluated microbial water quality in relation to the application of rapid molecular methods for analysis of highly pathogenic bacteria such as *Mycobacterium* spp., *Vibirio* spp., *Listeria monocytogenes*, *Clostridium perfringens*, *Clostridium botulinum*, *Shigella* spp., *Salmonella* spp., and *Yersinia enterocolitica*.

Nontuberculosis mycobacteria (NTM) are free-living saprophytes that are found in water, soil, animals and dairy products. NTM were not accepted as human pathogens until the 1950s, but human pathogenicity is now well recognized [4]. More than 150 NTM species have been described and new species continue to be identified [5]. In the last three decades it was confirmed that these bacteria are opportunistic pathogens for humans, animals, poultry and fish [5]. The main feature of mycobacterial cells which is responsible for their survival and wide distribution is the presence of a lipid-rich outer membrane. This hydrophobic membrane is responsible for adaptation, surface adherence and biofilm formation. The outer membrane is also responsible for antibiotic and disinfectant resistance. All mycobacterial species are able to grow at low carbon levels, a feature allowing them to survive, persist, grow and form biofilms in a low level nutrient environment such as drinking water [5]. Water is currently not routinely tested for mycobacteria and detection methods based on culturing require several days to get the results. In addition, water is often only tested retrospectively when other signs of contamination are present to alert the public when potentially already exposed to the health hazard. There is therefore a need for a rapid, inexpensive and sensitive method for the detection of mycobacteria from environmental sources.

Methods based on the amplification of organism-specific nucleic acids are faster than culturing methods and yield high precision and sensitivity to reliably and simultaneously detect waterborne pathogens. The aim of the present work was to conduct a two-year monitoring study to the water quality from the Dam of Iskar and the Black Sea. We focused on the application of rapid molecular tests based on the use of PCR and Real-Time PCR for detection of the key-pathogens *Mycobacterium* spp., *Vibirio* spp., *Listeria monocytogenes*, *Clostridium perfringens*, *Clostridium botulinum*, *Shigella* spp., *Salmonella* spp., and *Yersinia enterocolitica*. No previous studies have been carried out in Bulgaria using molecular analysis of pathogens including the detection of mycobacteria.

## 2. Experimental Section 

### 2.1. Sampling 

**Figure 1:**
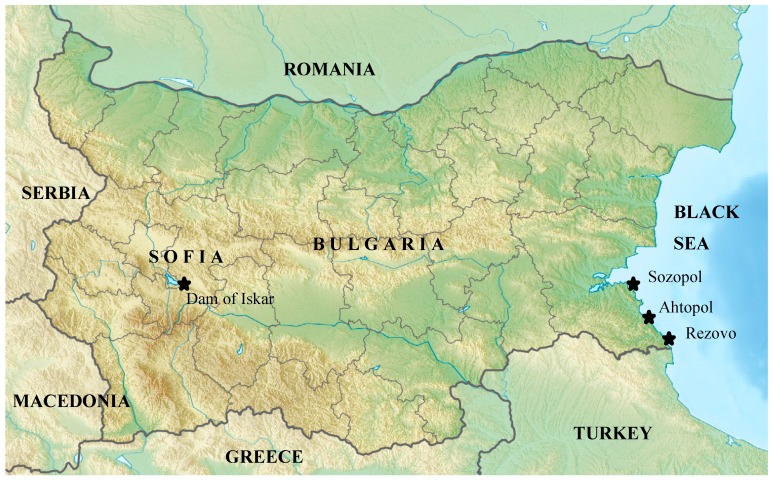
Map of Bugaria, Black Sea and the sampling sites. Sampling sites are highlighted with a 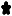.

The monitoring period was from June 2012 to February 2014, yielding a total of 38 water samples—24 from the Dam of Iskar (one sample per month) and 14 from the Black Sea coastal zone (one sample per two months) (Figure 1, Appendix, Table A1). All samples consisted of 50 L taken with a bucket at from the surface at 0 m deep, brought to the laboratory the same day and immediately filtered to concentrate microorganisms using an ultrafiltration device (dialyser); a 1.8 m^2^ sterile, disposable, polysulfone hollow-fiber module (Etropal JSC, Etropole, Bulgaria). Water was pushed at a constant 15 psi pressure through the hollow fibers using a peristaltic pump. After filtration, the microorganisms trapped within the filter fibers were recovered by backflushing in the reverse direction (from the outside to the inside of the fibers) with one liter of eluting solution (phosphate buffered saline 0.01 M (Sigma, St. Louis, MO, USA), Tween 80 0.5% (Sigma), sodium hexametaphosphate 0.01% (Sigma) and antifoam B 0.1% (Sigma). The eluted concentrate was preserved at 4 °C for immediate analyses or at −80 °C for longer storage. An aliquot of 200 mL of the eluted concentrate, corresponding to 10 L of the initial volume, was filtered through 20, 5, 2, 0.8 and 0.45 µM filters to further concentrate the sample. Each filter was processed as follows: the 0.45 µM filter was processed with 1 mL of lysis buffer (20 mg/mL lysozyme, Sigma); 20 nM Tris HCl, pH 8.0 (Sigma); 2 mM EDTA (Sigma); 1.2% Triton (Sigma)) for 1 h at 37 °C by shaking at 500 rpm. (Eppendorf Thermomixer comfort, Eppendorf AG, Hamburg, Germany). DNA was extracted from 200 µL. Six µL of Proteinase K 20 mg/mL (QIAGEN, GmbH, Hilden, Germany) were added and incubated at 56 °C for 2 h. Then, 150 µL of Magnetic Bead suspension (Perkin Elmer Inc, Waltham, MA, USA) were added. DNA was purified by processing the sample with Prepito device (Prepito, Chemagen AG, Baesweiler, Germany) and dissolved in 50 µL TE buffer following manufacture’s recommendation. Of this, 5 µL were used in duplicate Real-Time PCR analysis. The initial step allowed 50× sample concentration. The second step allowed additional 200× concentration, thus the final sample concentration was about 10^4^. From the 50× concentrate 40 mL were serially filtered as described above, including an end filtration through 0.1 µM filter for viral nucleic acids extraction.

### 2.2. Bacterial and Viral Detection

Fifty µL of the concentrated 50× water sample was stained by Ziehl-Neelsen and examined by microscopy for primary screening for mycobacteria [6]. Bacterial genomic DNA was extracted from the 0.45 µM filter which was processed with 1 mL of TRIZOL^TM^ reagent. 200 µL were used for DNA extraction applying a standard procedure [7]. All samples have been tested for 15 different pathogens including species of *Mycobacterium* spp., *Vibirio* spp., *Listeria monocytogenes*, *Campilobacter* spp., *Pseudomonas* spp., *Shigella* spp., *Salmonella* spp., pathogenic *Escherichia coli*, *Legionella* spp., *Yersinia enterocolitica and Rotaviruses.*

For the detection and identification of *Mycobacterium* spp., separate amplifications of three markers were applied. Amplification of the partial *rpoB* gene (360 bp) was performed using primers Rpo5′ (5′-TCAAGGAGAAGCGCTACGA-3′) and Rpo3′ (5′-GGATGTTGATCAGGGTCTGC-3′), as described by [8]. The second marker targeted was part of the 16S-23S spacer, amplified with primers Sp1 (5′-ACC TCC TTT CTA AGG AGC ACC-3′) and Sp2 (5′-GAT GCT CGC AAC CAC TAT CCA-3′) [9]. These primers amplified a 200–350 bp fragment specific to all mycobacteria The amplification was accomplished by an initial denaturation at 94 °C for 5 min, and 30 cycles at 94 °C for 30 seconds, 56 °C for 1 min and 72 °C for 40 seconds, followed by an extension at 72 °C for 10 min. The third marker was a species specific IS6110 for *M. tuberculosis* applying primers Tb294 (5′-GGACAACGCCGAATTGCGAAGGGC-3′) and Tb850 (5′-TAGGCGTCGGTGACAAAGGCCACG-3′) [10]. The selected markers have been widely used and tested for specificity and sensitivity [8,9,10]. Real-Time PCR tests for the detection of all other bacterial and viral markers were obtained from Primerdesign (Southampton, UK). Viral nucleic acid extraction (rotavirus) was performed by filtering 40 mL of ultrafiltered sample on 0.1 μM filter (Merck Millipore, Billerica, MA, USA). Viral DNA was extracted from 200 µL applying a standard procedure [11]. Internal amplification controls provided with the kits were added to each sample. Target bacteria were quantified by comparing the sample’s amplification curve to the standard curves (four consecutive 10-fold dilutions of 2 × 10^5^ copies/µL) of control DNA.

## 3. Results and Discussion

Microscopic and PCR analyses demonstrated that no mycobacterial were present in the water samples (Table 1). In six water samples originating from the Black Sea, *Vibrio* spp. were detected. Rotavirus A was also identified in four samples from the Dam of Iskar. The detection of rotaviruses in the samples from the Dam of Iskar was prevalent during the summer months. In our study the majority of the tested microbial water pathogens in both sampling sites were positive during summer and early autumn. In seven samples from both locations we identified toxigenic *Escherichia coli* with markers for *stx1* and *stx2* at concentrations ranging from 50–3 × 10^2^ copies/100 mL. The presence of *Norovirus* and enterohemorrhagic *E. coli* (EHEC) in recreational waters could be considered as an epidemiological risk for gastrointestinal illnesses [12]. To our knowledge, the origins of occasional cases of diarrhea along the Black Sea coast, typically occurring during the summer season, are associated with food contamination. There is no evidence that the Black Sea or Dam of Iskar waters could be a direct source of diarrhea infections associated to salmonellosis [13]. The detected concentrations are below the EU and Bulgarian standards for total coliforms; 900 cfu/100 mL, for recreational waters. In four additional samples there were no detectable amounts of *Cryptosporidium* in two samples from the Black Sea and two samples from the Dam of Iskar using immunomagnetic separation and fluorescence microscopy (Black Sea: <0.01 oocysts/10 L; Dam of Iskar: <0.01 oocysts/10 L) [14]. Cryptosporidiosis is a zoonotic disease, with *C. parvum* and *C. hominis* being associated most with human infection. Although industrial animal farming is not permitted around the Dam of Iskar, wildlife still provides a source of pathogens. Mass spectrometry analysis on the same four samples did not identify the presence of cyanobacterial toxins [15].

Routine monitoring of water is necessary for prevention of human and animal health. Pathogenic microorganisms occur in relatively low concentrations in surface waters. Often the contamination episode is not detected until the public shows symptoms of infection. Detection of indicator bacteria of known pathogens in the water indicates potential presence of contamination. *E. coli* has been chosen as biological indicator of water safety, and is part of drinking water regulations (EU Council directive 98/83/EC on the quality of water intended for human consumption). *E. coli* is also used as a fecal pollution indicator for recreational bathing waters in Europe (Directive 2006/7/EC concerning the management of bathing water quality and repealing Directive 76/160/EEC). The applied ultrafiltration approach allowed us to concentrate 50 L samples and decrease the filtration duration. The ultrafiltration system showed effective simultaneous collection of several microorganism types (viruses, bacteria, parasites, algae) from large volumes of water without clogging.

**Table 1:** Occurrence of targeted pathogens from environmental water obtained from the Black Sea and the Dam of Iskar during the two year monitoring campaign. The results are based on qPCR and regular PCR.

Target microorganism	Black Sea (n = 14)	Dam of Iskar (n = 24)
*Mycobacterium* spp.	0	0
*Vibirio* spp.	6	0
*Listeria monocytogenes*	0	0
*Campilobacter* spp.	0	0
*Pseudomonas* spp.	1	0
*Shigella* spp.	0	0
*Salmonella* spp.	0	0
*Legionella* spp.	0	1
*Yersinia enterocolitica*	0	0
*Clostridium perfringens*	0	0
*Clostridium botulinum*	0	0
*E. coli* EHEC stx1 and stx2	3	4
*Staphylococcus aureus*	0	0
*Aeromonas* spp.	0	0
Rotavirus A	0	4

The Black Sea basin has unique ecological characteristics [16]. The sea is almost closed and water exchange with the Mediterranean Sea is through the Bosphorus (Bosfor) Strait. The Black Sea is meromictic, characterized by an aerobic surface layer with a depth up to 100 m comprising only 13% of the total volume of the basin (salinity is 17–18‰), and a deep, anaerobic, more saline (22‰) layer up to 2245 m deep. The surface layer is supplied from rivers. Biodiversity is appreciably lower than seen in comparable North Atlantic waters [17]. A monitoring study [18] in 2007 at 23 stations of the bathing waters in Varna’s Black Sea coastal area confirmed that microbiological parameters including ‘total coliforms’, ‘faecal coliforms’ and ‘faecal streptococci’ were in compliance with the current EC Bathing Water Directive (76/EC/160) and exhibited good water quality characteristics. Similar results of generally good microbial status of the Georgian coastal zone were also reported [17]. Continuous monitoring of water samples for presence of potential pathogens, such as *Mycobacterium*, *Vibrio*, *Listeria* and *Clostridium* species, is essential especially during warm seasons. For many of the opportunistic pathogens targeted in this study, no internationally acceptable levels of the pathogens in fresh or marine water have been established.

## 4. Conclusions

During a two-year period in 2012–2014, we evaluated the water quality of the Black Sea near Bulgaria and the Dam of Iskar by applying molecular monitoring tests. Enteropathogenic *Vibrio*, *E. coli* and Rotavirus species have been detected at low concentration in seasonal summer water samples from the Dam of Iskar. The studied sampling sites along the Black Sea coast were not yet seriously threatened by these pathogenic microorganisms, in spite of the rapid development of recreation areas during the last years. No *Mycobacterium* spp. were detected in either the Black Sea or Dam of Iskar water samples during the two years. The sampling sites, during the two year period, were considered free of mycobacteria and safe for baths and industrial production of drinking tap water based on the presence of these pathogens. The application of rapid molecular tools for the detection of pathogens in water samples represents a valuable tool for monitoring to aid control and prevention of spread.

## Appendix

**Table A1:** List of water samples collected from the Black Sea and the Dam of Iskar between 26.06.2012–07.02.2014. Sampling coordinates and results of detected pathogens. NA—result not available; Black Sea—Rezovo village 41^o^58^’^57.76^”^ N 28^o^1^’^54.00^”^E; Black Sea—Ahtopol 42^o^5^’^57.51^”^ N27^o^56^’^38.54^”^E; Black Sea—Sozopol 42^o^25^’^05.50^”^ N 27^o^41^’^18.47^”^E; Dam of Iskar—Shtarkelovo gnezdo 42^o^27^’^28.79^”^N 23^o^33^’^31.71^”^E; Dam of Iskar—Zlo kuche 42^o^23^’^35.27^”^N 23^o^33^’^10.51.

№/Date	Location/GPS coordinates	*Yersinia enterocolitica*	*Vibirio* spp	*Listeria monocytogenes*	*Mycobactrium* spp.	*Salmonella* spp	*Shigella* spp.	*Campilobacter* spp.	*E. coli* *EHEC* *stx1 and stx2*	*Legionella* spp.	*Clostridium perfringens*	*Clostridium botulinum*	*S. aureus*	*Aeromonas* spp	*P.aeruginosa*	*Rotavirus A*
1. 16.01.2013	Dam of Iskar—Zlo kuche	NEG	POS	NEG	NEG	NEG	NEG	NEG	NEG	NEG	NEG	NEG	NEG	NEG	NA	NA
2. 09.06.2013	Dam of Iskar—Shtarkelovo gnezdo	NEG	NEG	NEG	NEG	NEG	NEG	NEG	NEG	NEG	NEG	NEG	NEG	NEG	NA	POS
3. 16.06.2013	Dam of Iskar—Zlokuche	NEG	NEG	NEG	NEG	NEG	NEG	NEG	NEG	NEG	NEG	NEG	NEG	NEG	NA	POS
4. 22.06.2013	Dam of Iskar—Zlokuche	NEG	POS	NEG	NEG	NEG	NEG	NEG	NEG	NEG	NEG	NEG	NEG	NEG	NA	POS
5. 29.06.2013	Dam of Iskar—Zlokuche	NEG	NEG	NEG	NEG	NEG	NEG	NEG	NEG	NEG	NEG	NEG	NEG	NEG	NA	NEG
6. 14.07.2013	Dam of Iskar—Shtarkelovo gnezdo	NEG	NEG	NEG	NEG	NEG	NEG	NEG	NEG	NEG	NEG	NEG	NEG	NEG	NA	NA
7. 21.07.2013	Dam of Iskar—Shtarkelovo gnezdo	NEG	NEG	NEG	NEG	NEG	NEG	NEG	NEG	NEG	NEG	NEG	NEG	NEG	NA	NEG
8. 28.07.2013	Dam of Iskar—Shtarkelovo gnezdo	NEG	NEG	NEG	NEG	NEG	NEG	NEG	NEG	NEG	NEG	NEG	NEG	NEG	NA	NA
9. 04.08.2013	Dam of Iskar—Shtarkelovo gnezdo	NEG	NEG	NEG	NEG	NEG	NEG	NEG	NEG	NEG	NEG	NEG	NEG	NEG	NA	NA
10. 18.08.2013	Dam of Iskar–Shtarkelovo gnezdo	NEG	NEG	NEG	NEG	NEG	NEG	NEG	NEG	NEG	NEG	NEG	NEG	NEG	NA	NA
11. 15.09.2013	Dam of Iskar—Shtarkelovo gnezdo	NEG	NEG	NEG	NEG	NEG	NEG	NEG	NEG	NEG	NEG	NEG	NEG	NEG	NA	NA
12. 23.09.2013	Dam of Iskar—Shtarkelovo gnezdo	NEG	NEG	NEG	NEG	NEG	NEG	NEG	POS stx1	NEG	NEG	NEG	NEG	NEG	NA	NA
13. 29.09.2013	Dam of Iskar—Shtarkelovo gnezdo	NEG	NEG	NEG	NEG	NEG	NEG	NEG	POS stx1	NEG	NEG	NEG	NEG	NEG	NA	NA
14. 04.10.2013	Dam of Iskar—Shtarkelovo gnezdo	NEG	NEG	NEG	NEG	NEG	NEG	NEG	POS stx1	NEG	NEG	NEG	NEG	NEG	NA	NEG
15. 13.10.2013	Dam of Iskar—Shtarkelovo gnezdo	NEG	NEG	NEG	NEG	NEG	NEG	NEG	NEG	NEG	NEG	NEG	NEG	NEG	NA	NEG
16. 20.10.2013	Dam of Iskar—Shtarkelovo gnezdo	NEG	NEG	NEG	NEG	NEG	NEG	NEG	NEG	NEG	NEG	NEG	NEG	NEG	NA	NA
17. 27.10.2013	Dam of Iskar—Shtarkelovo gnezdo	NEG	NEG	NEG	NEG	NEG	NEG	NEG	NEG	NEG	NEG	NEG	NEG	NEG	NA	NA
18. 03.11.2013	Dam of Iskar—Shtarkelovo gnezdo	NEG	NEG	NEG	NEG	NEG	NEG	NEG	NEG	NEG	NEG	NEG	NEG	NEG	NA	NA
19. 06.11.2013	Dam of Iskar—Shtarkelovo gnezdo	NEG	NEG	NEG	NEG	NEG	NEG	NEG	NEG	NEG	NEG	NEG	NEG	NEG	NA	NA
20. 10.11.2013	Dam of Iskar—Shtarkelovo gnezdo	NEG	NEG	NEG	NEG	NEG	NEG	NEG	NEG	NEG	NEG	NEG	NEG	NEG	NA	NA
21. 17.11.2013	Dam of Iskar—Shtarkelovo gnezdo	NEG	NEG	NEG	NEG	NEG	NEG	NEG	NEG	NEG	NEG	NEG	NEG	NEG	NA	NA
22. 24.11.2013	Dam of Iskar—Shtarkelovo gnezdo	NEG	NEG	NEG	NEG	NEG	NEG	NEG	NEG	NEG	NEG	NEG	NEG	NEG	NA	NA
23. 12.05.14	Dam of Iskar—Shtarkelovo gnezdo	NEG	NEG	NEG	NEG	NEG	NEG	NEG	NEG	NEG	NEG	NEG	NEG	NEG	NEG	POS
24. 30.06.14	Dam of Iskar—Shtarkelovo gnezdo	NEG	NEG	NEG	NEG	NEG	NEG	NEG	POS	POS	NEG	NEG	NEG	NEG	NEG	NEG
25. 09.09.2012	Black Sea—Rezovo village	NEG	POS	NEG	NEG	NEG	NEG	NEG	NEG	NEG	NEG	NEG	NEG	NEG	NA	NA
26. 16.09.2012	Black Sea—Ahtopol	NEG	POS	NEG	NEG	NEG	NEG	NEG	NEG	NEG	NEG	NEG	NEG	NEG	POS	NEG
27. 23.09.2012	Black Sea—Rezovo village	NEG	POS	NEG	NEG	NEG	NEG	NEG	NEG	NEG	NEG	NEG	NEG	NEG	NA	NEG
28. 12.01.2013	Black Sea—Sozopol	NEG	POS	NEG	NEG	NEG	NEG	NEG	NEG	NEG	NEG	NEG	NEG	NEG	NA	NA
29. 01.05.2013	Black Sea – Ahtopol	NEG	NEG	NEG	NEG	NEG	NEG	NEG	POS stx2	NEG	NEG	NEG	NEG	NEG	NA	NA
30. 23.06.2013	Black Sea—Rezovo village	NEG	NEG	NEG	NEG	NEG	NEG	NEG	NEG	NEG	NEG	NEG	NEG	NEG	NA	NA
31. 20.07.2013	Black Sea—Rezovo village	NEG	NEG	NEG	NEG	NEG	NEG	NEG	POS stx2	NEG	NEG	NEG	NEG	NEG	NA	NA
32. 31.08.2013	Black Sea—Rezovo village	NEG	NEG	NEG	NEG	NEG	NEG	NEG	NEG	NEG	NEG	NEG	NEG	NEG	NA	NA
33. 01.09.2013	Black Sea—Rezovo village	NEG	NEG	NEG	NEG	NEG	NEG	NEG	POS stx2	NEG	NEG	NEG	NEG	NEG	NA	NEG
34. 10.10.2013	Black Sea—Rezovo village	NEG	NEG	NEG	NEG	NEG	NEG	NEG	NEG	NEG	NEG	NEG	NEG	NEG	NA	NA
35. 14.04.2014	Black Sea—Rezovo village	NEG	POS	NEG	NEG	NEG	NEG	NEG	NEG	NEG	NEG	NEG	NEG	NEG	NA	NEG
36. 25.07.2014	Black Sea—Rezovo village	NEG	NEG	NEG	NEG	NEG	NEG	NEG	NEG	NEG	NEG	NEG	NEG	NEG	NA	NEG
37. 25.08.2014.	Black Sea—Rezovo village	NEG	NEG	NEG	NEG	NEG	NEG	NEG	NEG	NEG	NEG	NEG	NEG	NEG	NA	NEG
38. 05.09.2014	Black Sea—Rezovo village	NEG	POS	NEG	NEG	NEG	NEG	NEG	NEG	NEG	NEG	NEG	NEG	NEG	NA	NEG

## References

[1] Panaiotov S., Ciccozzi M., Brankova N.B., Levterova V.S., Mitova-Tiholova M., Amicosante M., Rezza G., Kantardjiev T.V. (2009). An outbreak of Q fever in Bulgaria. Ann. Ist. Super Sanita.

[2] Kantardjiev T., Ivanov I., Velinov T., Padeshki P., Popov B., Nenova R., Mincheff M. (2006). Tularemia outbreak, Bulgaria, 1997–2005. Emerg. Infect. Dis..

[3] Christova I., Velinov T., Kantardjiev T., Galev A. (2004). Tularemia outbreak in Bulgaria. Scand. J. Infect. Dis..

[4] Cassidy P.M., Hedberg K., Saulson A., McNelly E., Winthrop K.L. (2009). Nontuberculous mycobacterial disease prevalence and risk factors: A changing epidemiology. Clin. Infect. Dis..

[5] Falkinham J.O. (2009). Surrounded by mycobacteria: Nontuberculous mycobacteria in the human environment. J. Appl. Microbiol..

[6] Bachiyska E. (2005). Handbook for Microbiological Diagnosis of Tuberculosis.

[7] Kremer K., Arnold C., Cataldi A., Gutierrez M.C., Haas W.H., Panaiotov S., Skuce R.A., Supply P., van der Zanden A.G., van Soolingen D. (2005). Discriminatory power and reproducibility of novel DNA typing methods for *Mycobacterium tuberculosis* complex strains. J. Clin. Microbiol..

[8] Lee H., Park H.J., Cho S.N., Bai G.H., Kim S.J. (2000). Species identification of mycobacteria by PCR-restriction fragment length polymorphism of the rpoB gene. J. Clin. Microbiol..

[9] Roth A., Reischl U., Streubel A., Naumann L., Kroppenstedt R.M., Habicht M., Fischer M., Mauch H. (2000). Novel diagnostic algorithm for identification of mycobacteria using genus-specific amplification of the 16S–23S rRNA gene spacer and restriction endonucleases. J. Clin. Microbiol..

[10] Wilson S.M., McNerney R., Nye P.M., Godfrey-Faussett P.D., Stoker N.G., Voller A. (1993). Progress towards a simplified polymerase chain reaction and its application to diagnosis of tuberculosis. J. Clin. Microbiol.

[11] Xiang X., Qiu D., Hegele R.D., Tan W.C. (2001). Comparison of different methods of total RNA extraction for viral detection in sputum. J. Virol. Methods.

[12] Soller S.A., Bartrand T., Ashbolt N.J., Ravenscroft J., Wade T.J. (2010). Estimating the primary etiologic agents in recreational freshwaters impacted by human sources of faecal contamination. Waters Res..

[13] Petrov P., Asseva G., Parmakova K., Kantardjiev T. (2013). Analysis of human salmonelloses in Bulgaria, 2007–2011.

[14] Boots B. (2014). Personal communication.

[15] Greer B., McNamee S., Boots G., Cimarelli L., Guillebault D., Helmi K., Marcheggiani S., Panaiotov S., Breitenbach V., Akcaalan R., Medlin L., Elliott C., Campbell K. (2015). A survey of the prevalence of freshwater toxins in samples from six European countries using a validated UPLC-MS/MS procedure. Personal communication.

[16] Kideys A.E. (2002). Fall and rise of the Black Sea ecosystem. Science.

[17] Wilson J., Komakihidze A., Osadchaya T., Alyomov S., Romanov A., Tediashvili M. (2008). Evaluating ecological quality in the north-eastern Black Sea coastal zone. Marine Pollu. Bull..

[18] Simeonova A.K., Chuturkova R.Z., Bojilova V.B. (2010). Bathing water quality monitoring of Varna Black Sea coastal zone, Bulgaria. Water Res..

